# Improving Nowcasting of Intense Convective Precipitation by Incorporating Dual-Polarization Radar Variables into Generative Adversarial Networks

**DOI:** 10.3390/s24154895

**Published:** 2024-07-28

**Authors:** Pengjie Cai, He Huang, Taoli Liu

**Affiliations:** 1 School of Mathematics and Computer Science, Guangdong Ocean University, Zhanjiang 524088, China; caipengjie@stu.gdou.edu.cn; 2 School of Fisheries, Guangdong Ocean University, Zhanjiang 524088, China; huanghe@gdou.edu.cn

**Keywords:** precipitation nowcasting, dual-polarization radar, generative adversarial network, deep learning

## Abstract

The nowcasting of strong convective precipitation is highly demanded and presents significant challenges, as it offers meteorological services to diverse socio-economic sectors to prevent catastrophic weather events accompanied by strong convective precipitation from causing substantial economic losses and human casualties. With the accumulation of dual-polarization radar data, deep learning models based on data have been widely applied in the nowcasting of precipitation. Deep learning models exhibit certain limitations in the nowcasting approach: The evolutionary method is prone to accumulate errors throughout the iterative process (where multiple autoregressive models generate future motion fields and intensity residuals and then implicitly iterate to yield predictions), and the “regression to average” issue of autoregressive model leads to the “blurring” phenomenon. The evolution method’s generator is a two-stage model: In the initial stage, the generator employs the evolution method to generate the provisional forecasted data; in the subsequent stage, the generator reprocesses the provisional forecasted data. Although the evolution method’s generator is a generative adversarial network, the adversarial strategy adopted by this model ignores the significance of temporary prediction data. Therefore, this study proposes an Adversarial Autoregressive Network (AANet): Firstly, the forecasted data are generated via the two-stage generators (where FURENet directly produces the provisional forecasted data, and the Semantic Synthesis Model reprocesses the provisional forecasted data); Subsequently, structural similarity loss (SSIM loss) is utilized to mitigate the influence of the “regression to average” issue; Finally, the two-stage adversarial (Tadv) strategy is adopted to assist the two-stage generators to generate more realistic and highly similar generated data. It has been experimentally verified that AANet outperforms NowcastNet in the nowcasting of the next 1 h, with a reduction of 0.0763 in normalized error (NE), 0.377 in root mean square error (RMSE), and 4.2% in false alarm rate (FAR), as well as an enhancement of 1.45 in peak signal-to-noise ratio (PSNR), 0.0208 in SSIM, 5.78% in critical success index (CSI), 6.25% in probability of detection (POD), and 5.7% in F1.

## 1. Introduction

Strong convective precipitation is typically associated with disastrous weather phenomena, such as rainstorms and hurricanes, which frequently result in considerable economic losses and casualties. Strong convective weather is characterized by small spatial scales, short life cycles, and pronounced suddenness. Consequently, providing accurate nowcasting [1] poses a considerable challenge.

In recent years, the integration of polarimetric radar variables and numerical weather prediction (NWP) [2] has been intensively explored in the field of nowcasting applications. However, the numerical weather prediction conducted on supercomputer services [3,4] can merely provide weather forecasts of medium and small spatial scales within the upcoming few hours. Based on the continuity equation, pySTEPS [4] and DARTS [5] solve for future motion fields and future intensity residuals and generate future precipitation data iteratively. The aforementioned approach [4,5] incorporates deep learning. NowcastNet [6] employs multiple autoregressive models to fit the motion field and intensity residuals separately, generates the provisional forecasted data through an iterative evolution operator, and reprocesses the provisional forecasted data to obtain the final forecasted data via an encoder–decoder structure network. Compared with DGMR [3] of recurrent neural networks, NowcastNet reduces the computational cost; however, the simplistic iterative method accumulates more errors. In adversarial training, NowcastNet only utilizes the final forecasted data and observed data but neglects the provisional forecasted data. Hence, the limitations of NowcastNet are as follows: the autoregressive model is subject to the “regressing to the mean” problem; the iterative evolution process is prone to inevitable error accumulation; in adversarial strategy, the significance of the provisional forecasted data is disregarded, and the final forecasted data are regarded as the actual data.

The above-mentioned approaches [3,4,5,6] utilize precipitation data as the input data, and the precipitation data are acquired through the numerical extrapolation method of polarimetric radar variables [7]. Concerning various meteorological systems and distinct geographical regions, the numerical extrapolation method [7] exhibits diverse numerical expressions, and its accuracy is positively correlated with the volume of data. The key to precipitation forecasting is to predict the precipitation intensity in the future period, and the horizontal reflectivity ($Z_{H}$) of polarimetric radar variables can reflect the precipitation intensity. With the upgrade of the weather radar [8] to a dual-polarization radar [9,10,11], the polarization variables become six variables. The differential reflectivity ($Z_{DR}$) and the specific differential phase shift ($K_{DP}$) [12] provide additional precipitation information [11,13], and $Z_{DR}$ and $K_{DP}$ help ensure the reliability of the horizontal reflectivity ($Z_{H}$). Shi et al. [14] proposed a convolutional long short-term memory (ConvLSTM) network based on convolutional neural networks and recurrent neural networks. However, ConvLSTM has only a single input information and does not use multiple radar variables. Based on multimodal learning methods [15,16], Pan et al. [17] proposed the idea of using multiple radar information as input, utilized the UNet [18] structure that can easily adjust and use multiple inputs, and proposed FURENet. In this study, FURENet [17] generates the provisional forecasted data. However, FURENet is afflicted by the “regression to average” issue (where “regression to average” implies that the data generated by the autoregressive model are overly blurred). Hence, the structural similarity loss (SSIM loss) [19,20] is employed to mitigate this “regression to average” issue.

Based on NowcastNet, this study proposes an Adversarial Autoregressive Network (AANet). The generator of AANet is partitioned into FURENet and the Semantic Synthesis Model (SSM), while the discriminator of AANet is the Inception network [21]. FURENet directly generates the provisional forecasted data, and SSIM loss alleviates the “regression to average” issue. The Semantic Synthesis Model [22] introduces the multi-head self-attention mechanism [23] and introduces a global attention mechanism for the past observed data and the provisional forecasted data to prevent SSM from gradually disregarding the role of the past observed data during the spatial normalization process. In the adversarial strategy stage, the discriminator classifies both the provisional forecasted data and the final forecasted data as false, while categorizing the observed data as true. Its objective is to facilitate the concurrent enhancement of FURENet and SSM in adversarial training and to impel FURENet to generate more realistic and more similar generated data, as well as to urge SSM to produce increasingly realistic and increasingly similar generated data, thereby improving the nowcasting performance of the two stages.

## 2. Methods

The Adversarial Autoregressive Network (AANet) consists of two parts: generator and discriminator. The generator produces forecast data in two phases: In the first stage, FURENet [17] directly generates the provisional forecasted data; in the second stage, the Semantic Synthesis Model (SSM) reprocesses the provisional forecasted data and generates the final forecasted data. discriminator takes the provisional forecasted data, final forecasted data, and observational data as input data. In adversarial training strategy, it promotes the two-stage generator to generate more realistic and more similar generated data: The first-stage generator (FURENet) generates more realistic and more similar provisional generated data; the second-stage generator (SSM) uses the provisional generated data to generate more realistic and more similar final generated data.

### 2.1. Method for Provisional Forecasted Data Generation

This subsection utilizes mathematical expressions solely to simulate the process of generating the provisional forecasted data in NowcastNet [6] and to simulate the process of generating the provisional forecasted data in AANet. The aim is to compare the errors generated during the generation of the provisional forecasted data via these two approaches. For the sake of descriptive convenience, the approach through which FURENet [17] (the generator model of the first stage of AANet) generates forecasted data is termed as the autoregressive method (A), while the method by which NowcastNet generates the provisional forecasted data is named as the Evolutionary Method (E).

#### 2.1.1. Autoregressive Method

The autoregressive method [17] directly generates forecasted data, as depicted in Equation (1):

(1) $$X^{1:T}=C+\sum_{i=0}^{T_{0}}\left(\varphi^{i}X^{-T_{0}:0}\right)+\varepsilon$$

X is the “precipitation data”; *C*, and *φ* are the constant tensor; ε is the random error.

The error of the autoregressive method merely originates from the “regression to average” issue, rather than being accumulated through the iterative evolution operator [6].

#### 2.1.2. Evolutionary Method

The evolutionary method [6] generates the motion field and intensity residuals using two autoregressive models (as presented in Equation (1)) and subsequently produces the forecasted data through the evolutionary operator. As depicted in Equation (2),

(2) $$X^{i}=\left(V^{i}⊚X^{i-1}+S^{i}\right)+\varepsilon^{i},i\in \left[1,T\right]$$

X is the “precipitation data”; ε is the random error; *V* and *S* represent the motion field and intensity residuals, respectively; and ⊚ denotes the transfer symbol.

The forecasted data $X^{i}$ are acquired via the evolution operator [6]. By conducting *T* iterations of the cycle for Equation (2), the forecasted data $X^{1:T}$ can be obtained.

The evolutionary method is influenced by two sources of error: the ambiguity error resulting from the “regression to average” issue of the autoregressive model and the positional error that accumulates progressively in the iterative evolutionary process.

### 2.2. Adversarial Autoregressive Network

The core objective of this study is to optimize the nowcasting approach of convective precipitation through deep learning, thereby offering efficient meteorological services to various socio-economic sectors. The Adversarial Autoregressive Network (AANet) is a nowcasting model for convective precipitation. Based on NowcastNet, AANet integrates and enhances the generative adversarial network framework, refines the generation method of the provisional forecasted data, incorporates the multi-head attention mechanism and SSIM loss, and proposes a two-stage adversarial strategy.

**Figure 1:**
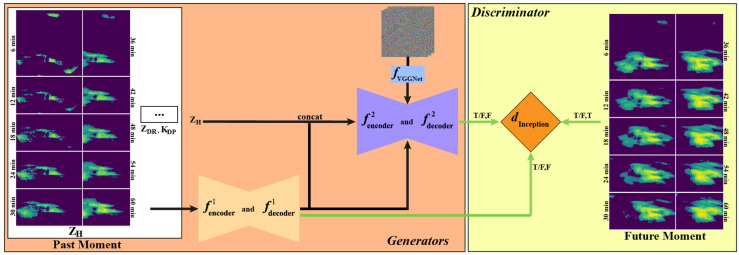
The process and schematic diagram of AANet.

Figure 1 depicts the process and schematic diagram of the Adversarial Autoregressive Network (AANet). Utilizing multiple polarized radar variables as the input, FURENet generates the provisional forecasted data, the Semantic Synthesis Model (SSM) generates the final forecasted data, and the discriminator is employed to distinguish observed data from the final forecasted data (as well as the provisional forecasted data).

#### 2.2.1. FURENet

FURENet adopts UNet [18] as its backbone network and utilizes the Squeeze-and-Excitation module (SE module) [24] to delay the fusion of features of multiple polarization variables. The Delayed Fusion Strategy [17,25,26,27] is usually applied to learning the complex relationships among various different kinds of information. When intricate relationships exist in different input variables, the input layer forcibly combines different input variables through linear operations, thereby causing the information entanglement effect. However, the Delayed Fusion Strategy can effectively address such issues. Let the tensor $\chi_{i}\in R^{N\times H\times W}$ represent a specific section of the polarized radar observation data (where *i* is *1*, *2*, and *3*, respectively, representing $Z_{H}$, $Z_{DR}$, and $K_{DP}$, *N* represents the time length, *H* represents the length, and *W* represents the width), and $f_{encoder}^{1}$ and $f_{decoder}^{1}$, respectively, represent the encoder features and decoder features of FURENet. The specific process of FURENet is shown in Equations (3) and (4).

(3) $$\left\{F_{n}^{i}|n=0,1,\ldots ,4\right\}=f_{encoder}^{1}\left(\chi_{i}^{1-T:0}\right),i=1,2,3$$

(4) $${\tilde{\chi_{1}}}^{1:T}=f_{decoder}^{1}\left(\left\{\left\{F_{n}^{i}|i=1,2,3\right\}|n=0,1,2,3,4\right\}\right)$$

$\chi_{i}^{0}$ denotes the data of the specific polarization variable at the present moment; $F_{n}^{i}$ represents the feature of the n-th-level semantic layer of the particular polarization variable data.

Equation (3): The encoder learns the features of the polarization variables of consecutive multiple frames and implements downsampling operations layer by layer semantically; the encoder outputs the semantic features ($F_{n}^{i}$) in each semantic layer and ultimately outputs the semantic feature sets ($\left\{F_{n}^{i}|n=0,1,\ldots ,4\right\}$). Equation (4): The decoder learns various top-level semantic features ($\left\{F_{n}^{i}|i=1,2,3,n=4\right\}$); by combining various same-level semantic features ($\left\{F_{n}^{i}|i=1,2,3,n=k\right\},k<4$), the decoder conducts upsampling on the semantic features; after multiple operations of layer semantics, the decoder obtains the provisional forecasted data (${\tilde{\chi_{1}}}^{1:T}$).

**Figure 2:**
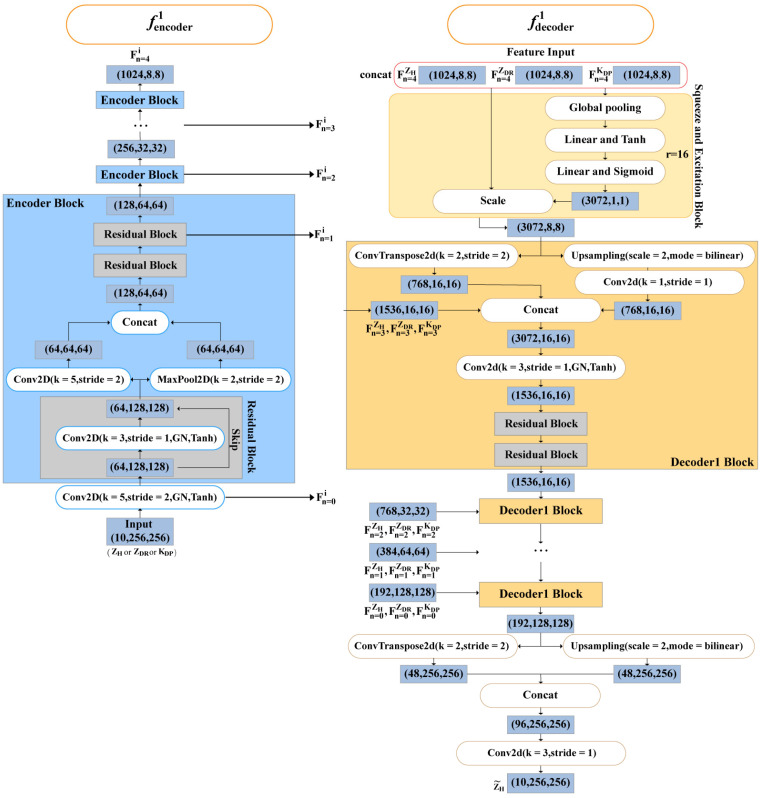
The illustration of the FURENet structure.

FURENet comprises three encoders and one decoder, and its structure is presented in Figure 2. The encoder consists of four encoding blocks and a convolutional layer (5 × 5 convolution kernel). The encoder blocks include a residual block [28], a bilateral downsampling, and two residual blocks. The bilateral downsampling includes a convolutional layer (with a 5 × 5 convolution kernel) and Maxpooling. The decoder is composed of the SE block, four decoder1 blocks, a bilateral upsampling, and a convolutional layer (5 × 5 convolution kernel). The bilateral upsampling includes Convtranspose [29] and bilinear interpolation [30]. The SE block comprises a global pooling layer, a linear layer, a Tanh layer, a linear layer, and a Sigmoid layer [31]. The decoder1 block is composed of a bilateral upsampling, a convolutional layer (3 × 3 convolutional kernel), and two residual blocks. The normalization functions and activation functions of the convolutional layer and the residual block are group normalization [32] and Tanh [33], respectively, except for the output layer.

#### 2.2.2. Semantic Synthesis Model

The Semantic Synthesis Model (SSM) consists of three parts, namely the encoder, the decoder, and the noise generator. The encoder encodes and learns the past observed data as well as the provisional forecasted data to acquire the data distribution characteristics ($F_{n=4}^{in}$). The decoder decodes the data distribution feature ($F_{n=4}^{in}$) and assigns it a global self-attention mechanism and a standardized affine transformation. Due to the influence of SPADE [22], the decoder will focus on the provisional predicted data and ignore the past observed data. The noise generator generates a learnable noise distribution feature (z) and cascades it with the data distribution feature ($F_{n=4}^{in}$), so the noise distribution feature (z) deepens the complex relationship between the provisional forecasted data and the past observed data. The noise generator uses VGGNet [34] as the backbone network. Let the tensor $\chi_{1}\in R^{N\times H\times W}$ be used to represent the observed data (where *N* represents the time length, *H* represents the length, and *W* represents the width), $\varepsilon \in R^{N\times H/4\times W/4}$ represents the random noise distribution, and $f_{encoder}^{2}$, $f_{VGGNet}$, and $f_{decoder}^{2}$ represent the encoder feature, noise generator feature, and decoder feature of the SSM, respectively. The specific process is shown in Equations (5)–(7) as follows:

(5) $$F_{n=4}^{in}=f_{encoder}^{2}\left(\chi_{1}^{1-T:0},{\tilde{\chi_{1}}}^{1:T}\right)$$

(6) $$F_{Z}=f_{VGGNet}\left(z\right),z∽N(0,1)$$

(7) $${\hat{\chi_{1}}}^{1:T}=f_{decoder}^{2}\left(\left\{F_{n=4}^{in},F_{Z}\right\}|{\tilde{\chi_{1}}}^{1:T}\right)$$

Equation (5): The encoder encodes and downsamples the past observation data ($\chi_{1}^{1-T:0}$) as well as the provisional forecasted data (${\tilde{\chi_{1}}}^{1:T}$), ultimately obtaining the data distribution feature ($F_{n=4}^{in}$). Equation (6): The noise generator encodes and downsamples Gaussian noise (with a mean of *0* and a variance of *1*), ultimately obtaining the noise distribution feature ($F_{Z}$). Equation (7): The data distribution characteristics and the noise distribution characteristics are cascaded; with the intervention of SPADE (where the feature normalization undergoes spatially affine transformation using the provisional forecasted data), the decoder decodes and upsamples the features and ultimately obtains the final forecasted data.

**Figure 3:**
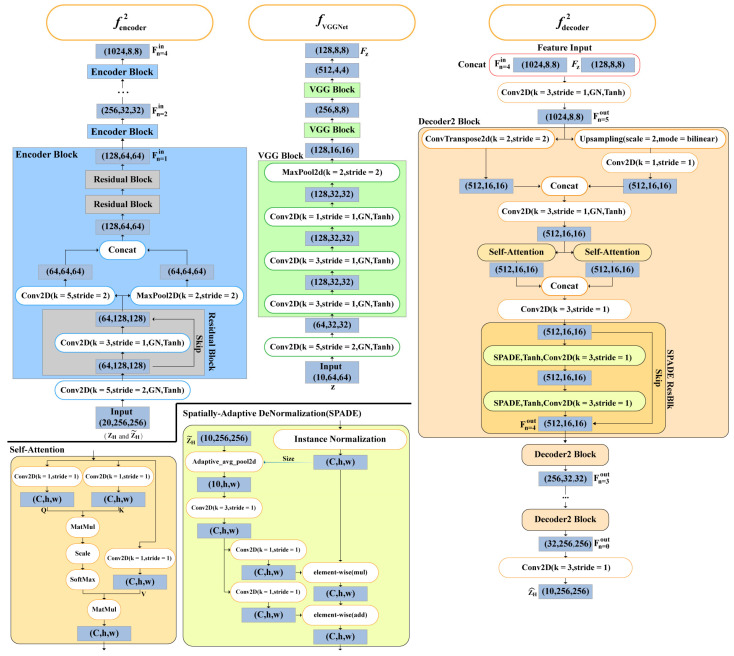
Schematic illustration of the SSM structure.

SSM consists of three parts, namely the encoder, the noise generator, and the decoder, and its specific structure is shown in Figure 3. The encoder consists of four encoder blocks and a convolutional layer (5 × 5 convolution kernel). The encoder block is composed of a residual block [28], a bilateral downsampling, and two residual blocks [28]. The normalization function is group normalization, and the activation function is Tanh. The decoder is composed of a convolutional layer (3 × 3 convolution kernels), five decoder2 blocks, and a convolutional layer (3 × 3 convolution kernels). The decoder2 block consists of bilateral upsampling, a convolutional layer (3 × 3 convolution kernels), a dual-head self-attention mechanism, a convolutional layer (3 × 3 convolution kernels) and a SPADE ResBlk [22]. The multi-head self-attention mechanism is composed of two self-attention [23] cascades. The ResBlk consists of two SPADEs and a Residual Skip. The principles of self-attention and spatially adaptive (de)normalization (SPADE [22]) will be presented subsequently.

The self-attention mechanism [23] is that the model reassigns the weights of features, meaning that the feature map has a global attention mechanism. Assume that the tensor ($X\in R^{C\times H\times W}$) represents the variable (where *C* represents the number of channels, *H* represents the length, and *W* represents the width), and the tensor ($\omega \in R^{C\times H_{\omega }\times W_{\omega }}$) represents the feature (with *C* representing the number of channels, $H_{\omega }$ representing the length, and $W_{\omega }$ representing the width), and the principle is depicted as shown in Equations (8)–(13).

(8) $$Q^{\left(c,h,w\right)}=\sum_{i=0}^{C-1}\sum_{j=0}^{H_{\omega }-1}\sum_{k=0}^{W_{\omega }-1}\left(\omega_{Q}^{\left(i,j,k\right)}X_{in}^{\left(i,h-\left\lfloor H_{\omega }/2\right\rfloor +j,w-\left\lfloor W_{\omega }/2\right\rfloor +k\right)}\right)$$

(9) $$K^{\left(c,h,w\right)}=\sum_{i=0}^{C-1}\sum_{j=0}^{H_{\omega }-1}\sum_{k=0}^{W_{\omega }-1}\left(\omega_{K}^{\left(i,j,k\right)}X_{in}^{\left(i,h-\left\lfloor H_{\omega }/2\right\rfloor +j,w-\left\lfloor W_{\omega }/2\right\rfloor +k\right)}\right)$$

(10) $$V^{\left(c,h,w\right)}=\sum_{i=0}^{C-1}\sum_{j=0}^{H_{\omega }-1}\sum_{k=0}^{W_{\omega }-1}\left(\omega_{V}^{\left(i,j,k\right)}X_{in}^{\left(i,h-\left\lfloor H_{\omega }/2\right\rfloor +j,w-\left\lfloor W_{\omega }/2\right\rfloor +k\right)}\right)$$

(11) $$\alpha =\frac{QK^{T}}{\sqrt{C}},\left(\alpha^{\left(c_{1},c_{2}\right)}=\frac{\sum_{j=0}^{H-1}\sum_{k=0}^{W-1}Q^{\left(c_{1},j,k\right)}K^{\left(c_{2},j,k\right)}}{\sqrt{C}}\right)$$

(12) $$\hat{\alpha }=softmax\left(\alpha \right),\left({\hat{\alpha }}^{\left(c_{1},c_{2}\right)}=\frac{e^{\alpha^{\left(c_{1},c_{2}\right)}}}{\sum_{i=0}^{C-1}e^{\alpha^{\left(c_{1},i\right)}}}\right)$$

(13) $$X_{out}=\hat{\alpha }V,\left(X_{out}^{\left(c,h,w\right)}=\sum_{i=0}^{C-1}{\hat{\alpha }}^{\left(c,i\right)}V^{\left(i,h,w\right)}\right)$$

The length (*H*) and the width (*W*) are equal; $X^{\left(i,j,k\right)}$ denotes the element of the tensor (*X*); and (*i*,*j*,*k*) represents the coordinates.

The principle of the self-attention mechanism [23]: $f\left(Q,K,V\right)=softmax\left(\frac{QK^{T}}{\sqrt{d}}\right)V$, which d is the number of channels. Equations (8)–(10): the autoregressive model encodes and learns the feature weights to obtain three tensors, including query ($Q\in R^{C\times H\times W}$), key ($K\in R^{C\times H\times W}$), and value ($V\in R^{C\times H\times W}$). Equation (11): tensor *Q* is transformed into matrix *Q* ($R^{C\times (H\times W)}\to R^{C\times (H\times W)}$), and tensor *K* is also transformed into matrix *K* ($R^{C\times (H\times W)}\to R^{C\times (H\times W)}$); then, a matrix operation is performed on *Q* and $K^{T}$ and multiplied by factor $\frac{1}{\sqrt{C}}$ to obtain α ($R^{C\times C}$). Equation (12): the softmax function is applied to the first dimension of α to obtain $\hat{\alpha }$ ($R^{C\times C}$). Equation (13): tensor *V* is transformed into matrix *V* ($R^{C\times (H\times W)}\to R^{C\times (H\times W)}$); a matrix operation is carried out between $\hat{\alpha }$ and *V* to obtain *X* ($R^{C\times (H\times W)}$).

The principle of SPADE [22]: Under the intervention of semantic conditions, a spatial affine transformation is carried out for normalization. SPADE reprocesses instance normalization [35]: the spatial affine transformation of normalization, which implements spatial adaptive adjustment for normalization under the intervention of semantic conditions. Suppose the tensor $X\in R^{C\times H\times W}$ is used to represent the variable (where *C* represents the number of channels, *H* represents the length, and *W* represents the width), and the tensor $\omega \in R^{C\times H_{\omega }\times W_{\omega }}$ is used to represent the feature (where *C* represents the number of channels, $H_{\omega }$ represents the length, and $W_{\omega }$ represents the width). The specific principle is detailed as shown in Equations (14)–(18).

(14) $$\mu^{\left(c\right)}=\frac{1}{H\times W}\sum_{i=0}^{H-1}\sum_{j=0}^{W-1}X_{in}^{\left(c,i,j\right)}$$

(15) $${\left(\sigma^{\left(c\right)}\right)}^{2}=\frac{1}{H\times W}\sum_{i=0}^{H-1}\sum_{j=0}^{W-1}{\left(X_{in}^{\left(c,i,j\right)}-\mu^{\left(c\right)}\right)}^{2}$$

(16) $$\gamma^{\left(c,h,w\right)}=\sum_{i=0}^{C-1}\sum_{j=0}^{H_{\omega }-1}\sum_{k=0}^{W_{\omega }-1}\left(\omega_{\gamma }^{\left(i,j,k\right)}X_{sc}^{\left(i,h-\left\lfloor H_{\omega }/2\right\rfloor +j,w-\left\lfloor W_{\omega }/2\right\rfloor +k\right)}\right)$$

(17) $$\beta^{\left(c,h,w\right)}=\sum_{i=0}^{C-1}\sum_{j=0}^{H_{\omega }-1}\sum_{k=0}^{W_{\omega }-1}\left(\omega_{\beta }^{\left(i,j,k\right)}X_{sc}^{\left(i,h-\left\lfloor H_{\omega }/2\right\rfloor +j,w-\left\lfloor W_{\omega }/2\right\rfloor +k\right)}\right)$$

(18) $$X_{out}^{\left(c,i,j\right)}=\gamma^{\left(c,h,w\right)}\frac{X_{in}^{\left(c,i,j\right)}-\mu^{\left(c\right)}}{\sigma^{\left(c\right)}}+\beta^{\left(c,h,w\right)}$$

The length *H* and the width *W* are equal; $X^{\left(i,j,k\right)}$ denotes the element of the tensor *X*, and (*i*,*j*,*k*) represents the coordinates; $X_{sc}$ denotes the semantic condition; μ denotes the mean value, and σ denotes the standard deviation.

Equations (14) and (15): the feature maps acquire their mean and standard deviation via specific formulas. Equations (16) and (17): The semantic conditions are encoded and learned through the autoregressive model in order to acquire the weight tensor *γ* and the bias tensor *β*. Equation (18): the normalization of the feature map employs a spatial affine transformation.

### 2.3. Training Principles

AANet is a Generative Adversarial Network [36], where the generator generates data, and the discriminator distinguishes between true and false data. The training principle of the Generative Adversarial Network: the generator and discriminator engage in a zero-sum game. With the continuous improvement of the vividness of the data generated by the generator, the discriminator must enhance its ability to distinguish between true and false. As the discriminator’s ability to distinguish true and false data becomes increasingly stronger, the generator has to improve its counterfeiting level.

The generator of AANet comprises two components. FURENet is accountable for generating the provisional forecasted data, while the Semantic Synthesis Model (SSM) generates the final forecasted data with the aid of the provisional forecasted data. The provisional forecasted data are helpful to enhance the authenticity and similarity of the final forecasted data. FURENet with better prediction performance indirectly improves the ability of SSM to generate forecasted data (it should be noted that FURENet and SSM are gradient-separated). Therefore, the discriminator deems the forecasted data (including the provisional and the final ones) as false data, while considering the observed data as true data.

For ease of description, let tensor $Y\in R^{B\times T\times H\times W}$ represent the observation data (*B* represents Batchsize, *T* represents the number of time steps, *H* represents the length, and *W* represents the width), and tensor $X\in R^{B\times T\times H\times W}$ represents the forecast data ($X_{1}$ represents the provisional forecasted data, and $X_{2}$ represents the final forecasted data).

#### 2.3.1. Mean Square Error Loss

To reduce the error distribution between generated and observed data, the mean square error loss function was used to calculate the error distribution between generated and observed data. Considering that the generator is a two-stage model, it is indispensable to minimize the error distribution of the data generated in the two-stage process.

(19) $$J_{MSE}=\frac{1}{T\times H\times W}\sum_{i=0}^{B-1}\sum_{j=0}^{T-1}\sum_{k=0}^{H-1}\sum_{l=0}^{W-1}\left({\left(Y^{\left(i,j,k,l\right)}-X_{1}^{\left(i,j,k,l\right)}\right)}^{2}+{\left(Y^{\left(i,j,k,l\right)}-X_{2}^{\left(i,j,k,l\right)}\right)}^{2}\right)$$

$X^{\left(i,j,k,l\right)}$ denotes the elements of the tensor *X*, and (*i*,*j*,*k*,*l*) represents the coordinates.

#### 2.3.2. Structural Similarity Loss

The autoregressive model suffers from the “regression to average” issue, namely, the generated data exhibits a propensity to converge towards the average value. SSIM Loss effectively alleviates the influence resulting from the “regression to average” issue and significantly enhances the similarity between the generated data and the observed data. The principle of SSIM [19,20] is shown in Equation (20) as follows:

(20) $$SSIM\left(x,y\right)=\frac{\left(2\mu_{x}\mu_{y}+c_{1}\right)\left(2\sigma_{xy}+c_{2}\right)}{\left(\mu_{x}^{2}+\mu_{y}^{2}+c_{1}\right)\left(\sigma_{x}^{2}+\sigma_{y}^{2}+c_{2}\right)}$$

$\mu_{x}$ and $\mu_{y}$ are the mean values of *x* and *y*, respectively; $\sigma_{x}$ and $\sigma_{y}$ are the standard deviations of *x* and *y*, respectively; $\sigma_{xy}$ is the covariance of *x* and *y*; the constants are taken as $K_{1}=0.01$, $K_{2}=0.03$, and *L* indicates the size of the range of values, so $c_{1}={\left(K_{1}L\right)}^{2}$, $c_{2}={\left(K_{2}L\right)}^{2}$.

For SSIM Loss, it should be applied locally rather than globally. The standard deviation, and covariance were computed by a sliding convolution (with a window size of *T × 11 × 11*). The sliding convolution is the Gaussian weighted tensor ($w\in R^{T\times 11\times 11}$) with circular symmetry characteristics (the standard deviation of *w* is *1.5*, and the total value is $1=\sum_{j=0}^{T-1}\sum_{k=0}^{11-1}\sum_{l=0}^{11-1}w^{\left(j,k,l\right)}$). Let $\mu \in R^{B\times R\times C}$ represent the mean (*B* represents batchsize, *R* represents the number of rows, and *C* represents the number of columns), and σ represents the standard deviation (covariance), as shown below:

(21) $$\mu_{F}^{\left(b,r,c\right)}=\sum_{t=0}^{T-1}\sum_{i=0}^{11-1}\sum_{j=0}^{11-1}w^{\left(t,i,j\right)}F^{\left(b,t,11\times r+i,11\times c+j\right)},F=X,Y$$

(22) $${\left(\sigma_{F}^{\left(b,r,c\right)}\right)}^{2}=\sum_{t=0}^{T-1}\sum_{i=0}^{11-1}\sum_{j=0}^{11-1}w^{\left(t,i,j\right)}{\left(F^{\left(b,t,11\times r+i,11\times c+j\right)}-\mu_{F}^{\left(b,r,c\right)}\right)}^{2},F=X,Y$$

(23) $$\sigma_{XY}^{\left(b,r,c\right)}=\sum_{t=0}^{T-1}\sum_{i=0}^{11-1}\sum_{j=0}^{11-1}w^{\left(t,i,j\right)}\left(X^{\left(b,t,11\times r+i,11\times c+j\right)}-\mu_{X}^{\left(b,r,c\right)}\right)\left(Y^{\left(b,t,11\times r+i,11\times c+j\right)}-\mu_{Y}^{\left(b,r,c\right)}\right)$$

(24) $$L_{SSIM}\left(X,Y\right)=\sum_{b=0}^{B-1}\sum_{r=0}^{R-1}\sum_{c=0}^{C-1}\left(1-\frac{\left(2\mu_{X}^{\left(b,r,c\right)}\mu_{Y}^{\left(b,r,c\right)}+c_{1}\right)\left(2\sigma_{XY}^{\left(b,r,c\right)}+c_{2}\right)}{\left({\left(\mu_{X}^{\left(b,r,c\right)}\right)}^{2}+{\left(\mu_{Y}^{\left(b,r,c\right)}\right)}^{2}+c_{1}\right)\left({\left(\sigma_{X}^{\left(b,r,c\right)}\right)}^{2}+{\left(\sigma_{Y}^{\left(b,r,c\right)}\right)}^{2}+c_{2}\right)}\right)$$

(25) $$J_{SSIM}=\frac{1}{R\times C}\left(L_{SSIM}\left(X_{1},Y\right)+L_{SSIM}\left(X_{2},Y\right)\right)\;$$

$X^{\left(i,j,k,l\right)}$ denotes the elements of the tensor *X*, and (*i*,*j*,*k*,*l*) represents the coordinates.

Equations (21)–(23): local feature maps calculate the mean tensor, standard deviation tensor, and covariance tensor through circular-symmetric Gaussian weighted tensors. Equations (24) and (25): firstly, the mean tensor, standard deviation tensor, and covariance tensor are employed to compute the local SSIM value; subsequently, the local SSIM value is subtracted from 1 to obtain the local SSIM Loss; eventually, the average is computed based on all the local SSIM Loss.

#### 2.3.3. Two-Stage Adversarial Loss

**Figure 4:**
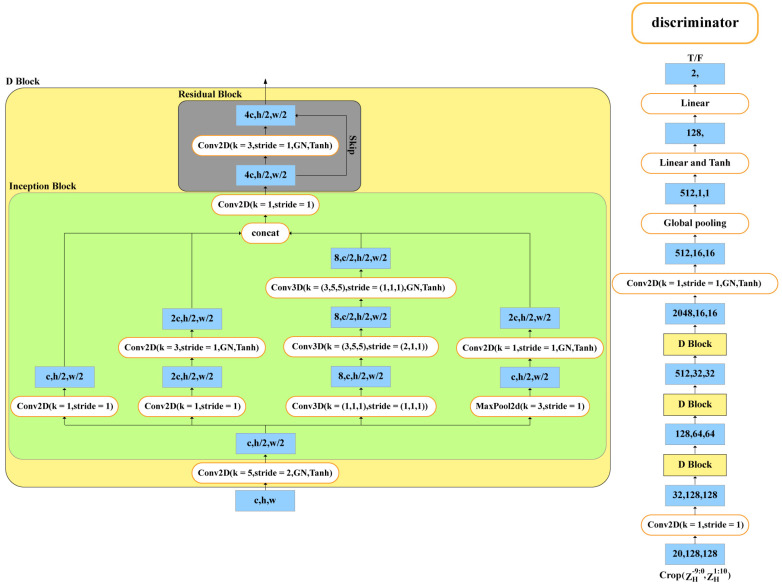
Schematic diagram of the structure of the discriminator.

The discriminator distinguishes between true and false data. The discriminator is composed of a convolutional layer (1 × 1 convolution kernel), four D blocks, a convolutional layer (1 × 1 convolution kernel), a global pooling layer, and two linear layers, as detailed in Figure 4. The D block has a convolutional layer (5 × 5 convolutional kernels), an inception block [21], and a residual block [28]. The inception block has four branches such as jump connections, spatial features, spatiotemporal features, and pooling features: the jump connections are the 2D convolutional layer (1 × 1 convolutional kernel); the spatial features are the 2D convolutional layer (1 × 1 convolutional kernel), and the 2D convolutional layer (3 × 3 convolutional kernels); the spatiotemporal features are the 3D convolutional layer (1 × 1 × 1 convolutional kernel), and two 3D convolutional layers (3 × 5 × 5 convolutional kernels); the pooling features are the 2D convolutional layer (1 × 1 convolutional kernel) and Maxpool. The normalization and activation functions are Group Normalization and Tanh, respectively. Observations, provisional forecasts, and final forecasts are used as discriminator input data, with observations labeled 1 and provisional and final forecasts labeled 0. In the input data, the ratio of observed data to final forecasted data (and provisional forecasted data) is one.

Suppose $G_{1}$ represents the first-stage model (FURENet) of the generator, $G_{2}$ represents the second-stage model (SSM) of the generator, *D* represents the discriminator, $p_{data}$ represents the random radar data distribution ($X^{1-T:0}$ represents the past observation data, and $X^{1:T}$ represents the future observation data), and $p_{noise}\left(z\right)$ represents Gaussian noise. The two-stage adversarial strategy is presented as follows:

(26) $$\begin{array}{l} \underset{G_{1}}{\min}\underset{G_{2}}{\min}\underset{D}{\max}V\left(D,G_{1},G_{2}\right)=E_{X^{1:T}∽p_{data}\left(X^{1-T:T}\right)}\log D\left(X^{1-T:T}\right)\\ \;+E_{X^{1-T:0}∽p_{data}\left(X^{1-T:T}\right)}\log\left(1-D\left(G_{1}\left(X^{1-T:0}\right),X^{1-T:T}\right)\right)\\ \;+E_{\begin{array}{c} X^{1-T:0}∽p_{data}\left(X^{1-T:T}\right)\\ z∽p_{noise}\left(z\right) \end{array}}\log\left(1-D\left(G_{2}\left(G_{1}\left(X^{1-T:0}\right),z\right),X^{1-T:T}\right)\right) \end{array}$$

Equation (26): Try to find the optimal discriminator *D* that maximizes the likelihood of distinguishing between true data and false data; try to find the optimal generator $G_{2}$ that minimizes the likelihood that the generated data will be recognized by the discriminator; try to find the optimal generator $G_{1}$ that minimizes the likelihood that the generated data will be recognized by the discriminator. In a zero-sum game, the generator tries to cheat the discriminator: generator $G_{2}$ boosts the faking ability and therefore generates more realistic fake data; based on the increased faking ability, generator $G_{1}$ also generates more realistic faking data, so generator $G_{2}$ receives better faking material and generates more realistic faking data. The specific application differs from the above theory, as described in Algorithm 1.
**Algorithm 1.** The detailed process explanation of the two-stage adversarial strategy.Input: past observations $Y_{1}$, future observations $Y_{2}$, provisional forecasts $X_{1}$, final forecasts $X_{2}$, which the shape of the input data is $R^{B\times T\times H\times W}$.1: Four random cropping operations ($R^{2B\times T\times H\times W}\to R^{2B\times T\times \frac{H}{2}\times \frac{W}{2}}$) are performed on $\left(Y_{1},Y_{2}\right)$. It is assigned the label 1, and concatenated along the first dimension;$p_{y}=\left\{\left(y_{\left(0,0\right)}^{2T\times \frac{H}{2}\times \frac{W}{2}},1\right),...,\left(y_{\left(0,3\right)}^{2T\times \frac{H}{2}\times \frac{W}{2}},1\right),...,\left(y_{\left(B-1,3\right)}^{2T\times \frac{H}{2}\times \frac{W}{2}},1\right)\right\}$2: Two random cropping operations ($R^{2B\times T\times H\times W}\to R^{2B\times T\times \frac{H}{2}\times \frac{W}{2}}$) are performed on $\left(Y_{1},X_{1}\right)$. It is assigned the label 1, and concatenated along the first dimension;$p_{x_{1}}=\left\{\left({x_{1}}_{\left(0,0\right)}^{2T\times \frac{H}{2}\times \frac{W}{2}},0\right),\left({x_{1}}_{\left(0,1\right)}^{2T\times \frac{H}{2}\times \frac{W}{2}},0\right),...,\left({x_{1}}_{\left(B-1,1\right)}^{2T\times \frac{H}{2}\times \frac{W}{2}},0\right)\right\}$3: Two random cropping operations ($R^{2B\times T\times H\times W}\to R^{2B\times T\times \frac{H}{2}\times \frac{W}{2}}$) are performed on $\left(Y_{1},X_{2}\right)$. It is assigned the label 1, and concatenated along the first dimension;$p_{x_{2}}=\left\{\left({x_{2}}_{\left(0,0\right)}^{2T\times \frac{H}{2}\times \frac{W}{2}},0\right),\left({x_{2}}_{\left(0,1\right)}^{2T\times \frac{H}{2}\times \frac{W}{2}},0\right),...,\left({x_{2}}_{\left(B-1,1\right)}^{2T\times \frac{H}{2}\times \frac{W}{2}},0\right)\right\}$4: Connect $p_{y}$, $p_{x_{1}}$, and $p_{x_{2}}$ and randomly sample m times;$\left(s,k\right)∼\left(p_{y},p_{x_{1}},p_{x_{2}}\right),\;sample\;4B\;times$5: Calculating cross-entropy loss.$J_{Tadv}=-\sum_{i=0}^{4B-1}\left(k\log_{10}D\left(s\right)+\left(1-k\right)\log_{10}\left(1-D\left(s\right)\right)\right)$

#### 2.3.4. Total Loss

The generator and the discriminator engage in a performance race, and the total loss function allows the generator and the discriminator to develop in adversity, with both models becoming more and more superior in performance.

(27) $$L=\alpha J_{MSE}+\beta J_{SSIM}+J_{Tadv}$$

The parameters *α* and *β* are constants. In the experiment, *α* and *β* are, respectively, 0.5; nevertheless, setting *α* to 1 and *β* to 4 produces better results.

## 3. Results

### 3.1. Implementation Details

The “NJU_CPOL_update2308” dataset is the data for the 20th “Huawei Cup” China Graduate Student Mathematical Modeling Contest. This dataset is the test data of Problem F of “Nowcasting of Severe Convective Precipitation”. The continuous precipitation process is represented by using three different continuous multi-frame grid data ($Z_{H}$, $Z_{DR}$, and $K_{DP}$). The time interval between two adjacent frames of data is 6 min, and this interval is also the scanning interval of the dual-polarization radar. The shape of the grid data is 256 × 256, and the corresponding plane area is 256 km × 256 km. The NJU_CPOL_update2308 dataset encompasses a considerable quantity of invalid data. Hence, manual screening is imperative, and radar data quality control [37] (encompassing data alignment, outlier elimination, removal of non-meteorological echoes, and data filtering) should be executed. The initial size of the NJU_CPOL_update2308 dataset was approximately 89 G. After manual screening and radar data quality control, the size of the dataset was reduced to 25.5 G.

The deep learning framework is pytorch, with the optimizer using AdamW and learning rate strategy using CosineAnnealingLR. The learning rate of the first-stage model of the generator is set at $4\times 10^{-4}$. The learning rate of the second-stage model of the generator is set at $4\times 10^{-5}$. The learning rate of the discriminator is set at $4\times 10^{-4}$. The graphics processor uses NVIDIA GeForce RTX 3090, which has 24 G of video memory. In the contrast model, the optimizer employs AdamW, and the learning rate strategy implements CosineAnnealingLR. The learning rate of the compared model is consistent with that of this study. Based on the use of the pre-trained model, the number of training iterations is 100.

### 3.2. Forecast Measurement Indicators

In this study, eight forecast measurement indicators such as normalized error (NE), root mean square error (RMSE), peak signal-to-noise ratio (PSNR), structural similarity (SSIM), critical success index (CSI), probability of detection (POD), F1, and false alarm ratio (FAR) are used to measure the excellence of the model. NE and RMSE describe the error of the forecasted data concerning the observed data. PSNR is used to represent the quality of the predicted data. SSIM describes the degree of similarity between the forecasted data and the observed data, with a value of 1 indicating the same and a value of −1 indicating not at all. CSI, POD, and F1 delineate the probability of success for the forecast. The FAR describes the probability of forecasted failure. Assuming that the tensor $Y\in R^{T\times H\times W}$ represents the observed data (*T* represents the number of time steps, *H* represents the length, and *W* represents the width), and the tensor $X\in R^{T\times H\times W}$ represents the forecasted data, the formulas for NE, RMSE, PSNR, SSIM, CSI, POD, F1, and FAR are given as follows (detailed formulas for SSIM are given in Equations (20)–(23), with the Batchsize set at 1):

(28) $$NE=\frac{\sum_{t=0}^{T-1}\sum_{h=0}^{H-1}\sum_{w=0}^{W-1}\left(Y^{\left(t,h,w\right)}-X^{\left(t,h,w\right)}\right)}{\sum_{t=0}^{T-1}\sum_{h=0}^{H-1}\sum_{w=0}^{W-1}Y^{\left(t,h,w\right)}}$$

(29) $$RMSE=\sqrt{\frac{1}{T\times H\times W}\sum_{t=0}^{T-1}\sum_{h=0}^{H-1}\sum_{w=0}^{W-1}{\left(Y^{\left(t,h,w\right)}-X^{\left(t,h,w\right)}\right)}^{2}}$$

(30) $$PSNR=20\log_{10}\frac{65}{RMSE}$$

(31) $$SSIM\left(X,Y\right)=\frac{\left(2\mu_{X}\mu_{Y}+c_{1}\right)\left(2\sigma_{XY}+c_{2}\right)}{\left(\mu_{X}^{2}+\mu_{Y}^{2}+c_{1}\right)\left(\sigma_{X}^{2}+\sigma_{Y}^{2}+c_{2}\right)}$$

(32) $$CSI=\frac{TP}{TP+FP+FN}$$

(33) $$POD=\frac{TP}{TP+FN}$$

(34) $$F1=\frac{2TP}{2TP+FP+FN}$$

(35) $$FAR=\frac{FP}{TP+FP}$$

$X^{\left(i,j,k\right)}$ denotes the elements of the tensor *X*, and (*i*,*j*,*k*) represents the coordinates; *TP* indicates that positive examples are true; *FP* indicates that negative examples are false; *FN* indicates that positive examples are false.

### 3.3. Ablation Experiments

To validate the performance of the model, AANet is compared with other models. The evolution method (E) of NowcastNet [6] generates the predicted data in two stages. Firstly, it uses the motion field and intensity residuals, and then, it uses the iterative evolution operator to generate the provisional forecasted data. The autoregressive method (A) of FURENet [17] is to generate provisional forecasted data directly. To validate the superiority of the evolutionary method (E) and the autoregressive method (A), these two methods were, respectively, implemented in FURENet and UNet. For the models that adopt the evolution method, their names are prefixed with “E”; for the models that utilize the autoregressive method, their names are prefixed with “A”. The evolutionary method (E) applies the objective function of NowcastNet [6] (except for the adversarial loss), and the autoregressive method (A) applies the objective of the function in this paper (except for the adversarial loss). To verify whether SSIM Loss can suppress the “regression to average” issue, comparative experiments with and without SSIM Loss are conducted on FURENet and UNet. To compare AANet and NowcastNet, the forecasted results were compared using AANet and NowcastNet. In order to verify the effectiveness of the two-stage adversarial strategy, AANet, AANet-adv (using the traditional adversarial loss), and AANet-noadv (not using the adversarial loss) are used for comparison. Finally, ablation experiments were conducted on AANet at each stage.

#### 3.3.1. Autoregressive Method and Evolutionary Method

**Figure 5:**
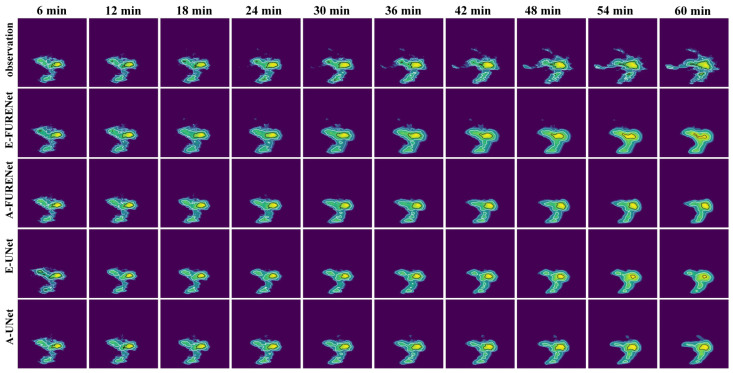
Visualization of the forecasted effect; the contour lines (light blue < white < black) are gradient lines of precipitation intensity.

**Figure 6:**
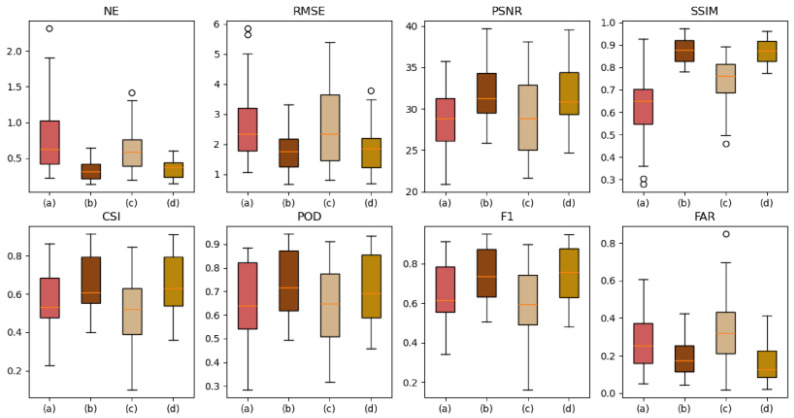
Box plot of model forecasted effect, among which (a), (b), (c), and (d) represent E-FURENet, A-FURENet, E-UNet, and A-UNet, respectively. The orange line represents the median line, and the black circle symbolizes the outliers.

**Figure 7:**
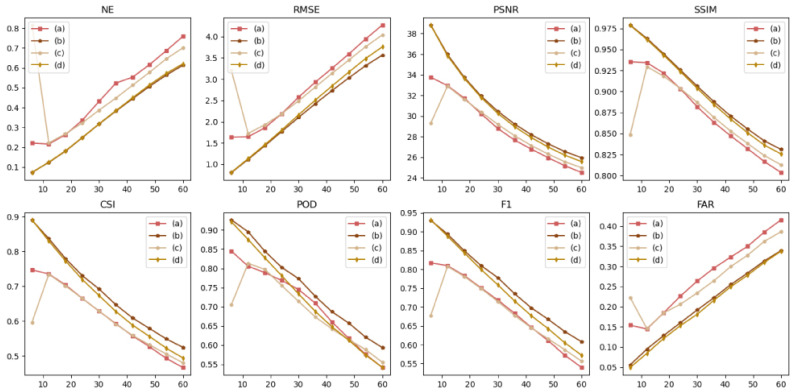
The forecasted effect at future moments, (a), (b), (c), and (d) represent E-FURENet, A-FURENet, E-UNet, and A-UNet, respectively.

The autoregressive method (A) and the evolutionary method (E) are forecasting methods. The autoregressive method (A) and evolutionary method (E) were used for FURENet and UNet, respectively, to verify the superiority of the autoregressive method (A) and evolutionary method (E). A-FURENet is the first-stage generator model of AANet; E-UNet is the first-stage generator model of NowcastNet, specifically as shown in Figure 5, Figure 6 and Figure 7, Table 1.

As can be seen in Figure 5, the autoregressive method (A) has a better forecasted performance and still captures more details in the larger future time. At smaller moments, the forecasted performance of the evolutionary method (E) and the autoregressive method (A) are comparable; as the time steps increase, the forecasting ability of the evolutionary method (E) decreases significantly, while the autoregressive method (A) still maintains a certain level of forecasting ability.

In the NE, RMSE, and FAR indicators, the autoregressive method (A) suppresses error better than the evolutionary method (E). In FURENet, the median line and variance of A-FURENet are smaller than the median line and variance of E-FURENet, and A-FURENet’s broken line is also below E-FURENet’s broken line, which is shown in Figure 6 and Figure 7. In UNet, the median line and variance of A-UNet are smaller than the median line and variance of E-UNet, and A-UNet’s broken line is also below E-UNet’s broken line, which is shown in Figure 6 and Figure 7. The increase in the NE value means that the model has the conditions of overfitting or underfitting. The increase in the RMSE value indicates that the deviation degree of the forecasted value from the true value is larger. The increase in the FAR value reveals that the probability of the model’s wrong prediction is higher. Compared to the evolutionary methods, the autoregressive methods suppress overfitting or underfitting, reduce the deviation between forecasted data and true data, and reduce the probability of false forecasts. The autoregressive method (A) lacks the evolution operator of the evolution method (E), in which the evolution operator generates the positional error accumulated by the iterative evolution process, so the autoregressive method (A) only has the error caused by the “regression to average” issue.

In the PSNR and SSIM indicators, the autoregressive methods (A) are better than the evolutionary methods (E). In FURENet, the median line of A-FURENet is higher than the median line of E-FURENet, the variance of A-FURENet is smaller than the variance of E-FURENet, and the broken line of A-FURENet is higher than the broken line of E-FURENet, which is shown in Figure 6 and Figure 7. In UNet, the median line of A-UNet is higher than the median line of E-UNet, the variance of A-UNet is smaller than the variance of E-UNet, and the broken line of A-UNet is higher than the broken line of E-UNet, shown in Figure 6 and Figure 7. A smaller value of PSNR indicates a more ambiguous prediction; a larger value of SSIM indicates that the forecasted data are more similar to the real data. The autoregressive method (A) provides more accurate and more similar forecasted data compared to the evolutionary method (E). The reason is that the error in the autoregressive method only stems from the “regression to average” issue, and there is no error accumulated due to the iterative evolution process.

In the CSI, POD, and F1 indicators, the autoregressive methods (A) are better than the evolutionary methods (E). In FURENet, the median line of A-FURENet is higher than the median line of E-FURENet, the variance of A-FURENet is smaller than the variance of E-FURENet, and the broken line of A-FURENet is higher than the broken line of E-FURENet, shown in Figure 6 and Figure 7. In UNet, the median line of A-UNet is higher than the median line of E-UNet, the variance of A-UNet is smaller than the variance of E-UNet, and the broken line of A-UNet is higher than the broken line of E-UNet (except for the broken line of POD), shown in Figure 6 and Figure 7. The larger the CSI value, the higher the prediction success rate of the model (considering the hit rate, false alarm rate, and missed alarm rate comprehensively); the larger the POD value, the higher the probability of the model’s prediction hit (considering the hit rate and missed alarm rate comprehensively); the larger the F1 value, the larger the POD and the smaller the FAR. The CSI and F1 of the autoregressive method (A) are greater than the CSI and F1 of the evolutionary method (E), but the POD of the autoregressive method (A) is comparable to the POD of the evolutionary method (E). The reason is that the evolutionary method (E) worsens the “re-gression to average” issue, which makes the prediction more ambiguous and leads to inflated values of POD.

As shown in Table 1, A-FURENet outperforms E-FURENet in forecast, with NE reduced by 0.1154, RMSE reduced by 0.557, PSNR improved by 2.05, SSIM improved by 0.0265, CSI improved by 7.16%, POD improved by 4.6%, F1 improved by 6.68%, and FAR reduced by 6.96%; the forecast of A-UNet is better than the forecast of E-UNet, with NE reduced by 0.142, RMSE reduced by 0.563, PSNR improved by 2.06, SSIM improved by 0.0293, CSI improved by 6.83%, POD improved by 3.46%, F1 improved by 6.25%, and FAR reduced by 6.52%. In FURENet and UNet models, the autoregressive method (A) provides similar performance enhancements for NE, RMSE, PSNR, SSIM, CSI, F1, and FAR. Still, the autoregressive method (A) provides a large difference in performance enhancement for POD. The autoregressive method (A) suffers from the “regression to average” issue, and the evolutionary method (E) exacerbates the “regression average” issue and leads to inflated POD values.

A-FURENet and E-UNet are the first-stage generators of AANet and NowcastNet, respectively, and A-FURENet has better performance with 0.1454 reduction in NE, 0.647 reduction in RMSE, 2.27 improvement in PSNR, 0.0322 improvement in SSIM, 8.44% improvement in CSI, 6.7% improvement in POD, 7.95% improvement in F1, and 5.89% reduction in FAR. It can be shown that the first-stage model of AANet’s generator outperforms the first-stage model of NowcastNet’s generator. In summary, the autoregressive method (A) accumulates less error in the forecasted process and has better forecasted performance.

#### 3.3.2. Comparative Experiments of Models with and without SSIM Loss

**Figure 8:**
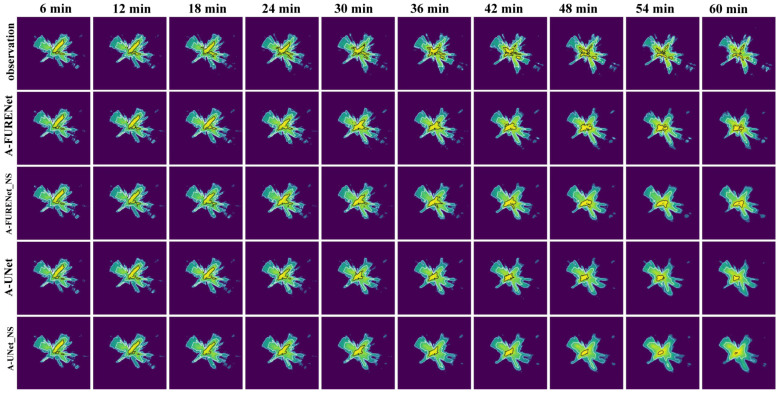
Visualization of the forecasted effect; the contour lines (light blue < white < black) are gradient lines of precipitation intensity.

**Figure 9:**
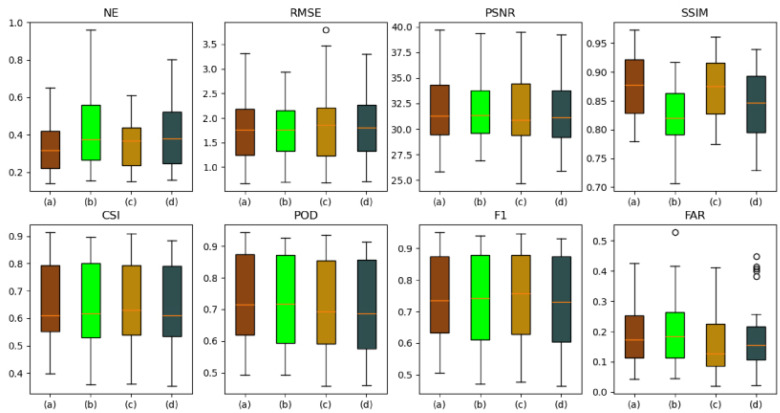
Box plot of model forecasted effect, among which (a), (b), (c), and (d) represent A-FURENet, A-FURENet_NS, A-UNet, and A-UNet_NS, respectively. The orange line represents the median line, and the black circle symbolizes the outliers.

**Figure 10:**
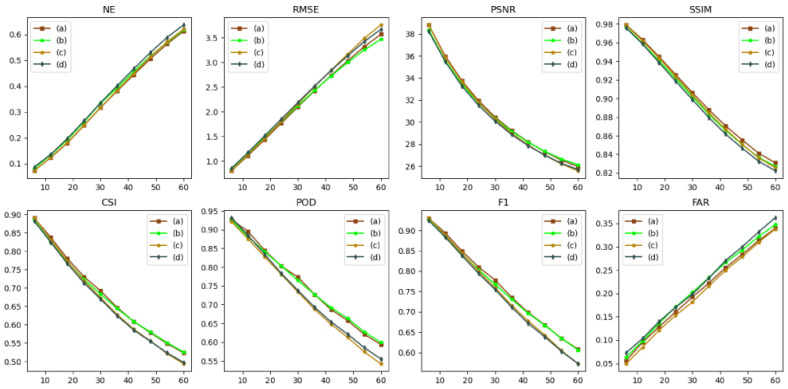
The forecasted effect at future moments, (a), (b), (c), and (d) represent A-FURENet, A-FURENet_NS, A-UNet, and A-UNet_NS, respectively.

To verify whether SSIM Loss can suppress the “regression to average” issue, comparative experiments with and without SSIM Loss are conducted on A-FURENet and A-UNet. As shown in Figure 8, Figure 9 and Figure 10, Table 2.

As can be seen in Figure 8, SSIM Loss gives the model the ability to capture more detail, effectively reducing the impact of the “regression to average” issue. At larger timescales, A-FURENet displays more data detail, but A-FURENet_NS (No SSIM Loss) uses the mean to override the data detail. Compared to A-UNet and A-UNet_NS, A-UNet_NS uses the mean to cover a large amount of detail, while A-UNet effectively suppresses the tendency to “regression to average”.

As shown in Box Figure 9, SSIM Loss has little effect on the model’s RMSE, PSNR, CSI, POD, F1, and FAR (excluding UNet’s FAR, the reason is that the presence of a large number of outliers), but SSIM Loss improves the model’s NE and SSIM performance significantly. Thus, SSIM Loss suppresses model overfitting or underfitting, and also facilitates the model to generate more realistic and more similar predictions. As shown in Broken Line Figure 10, SSLM Loss is only significant for the FAR of the model, and SSIM Loss has no significant effect on the other broken lines of the model. Therefore, SSIM Loss reduces the probability of model false forecasts. Based on the above phenomena, SSIM Loss suppresses the “regression to average” issue, suppresses model overfitting or underfitting, and reduces the ambiguity of the predictions, thus reducing the probability of false predictions.

As shown in Table 2, SSIM Loss acts positively on the model. For A-FURENet, NE decreased by 0.0107, RMSE decreased by 0.005, PSNR increased by 0.15, SSIM increased by 0.0044, CSI increased by 0.53%, POD increased by 0.15%, F1 increased by 0.57%, and FAR decreased by 0.97%. For A-UNet, NE decreased by 0.0162, RMSE decreased by 0.008, PSNR increased by 0.2, SSIM increased by 0.0044, CSI increased by 0.47%, POD decreased by 0.67%, F1 increased by 0.53%, and FAR decreased by 2.1%. In summary, SSIM Loss has a significant effect on the model’s FAR. SSIM Loss suppresses the “regression to average” issue, which reduces the ambiguity of the forecast and ultimately reduces the probability of false forecasts.

#### 3.3.3. AANet and NowcastNet

**Figure 11:**
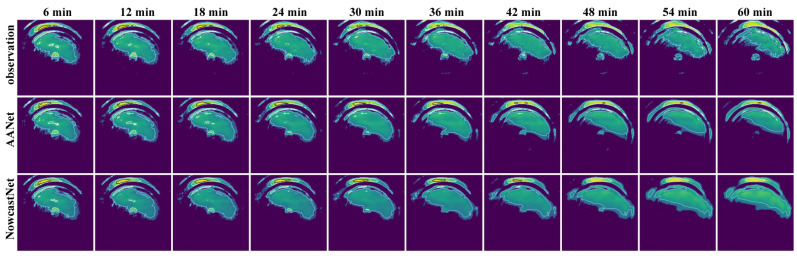
Visualization of the forecasted effect; the contour lines (light blue < white < black) are gradient lines of precipitation intensity.

**Figure 12:**
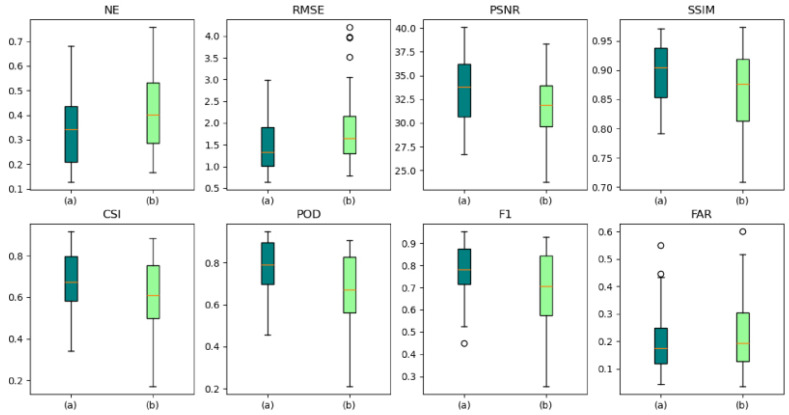
Box plot of model forecasted effect, among which (a), and (b) represent AANet, and NowcastNet, respectively. The orange line represents the median line, and the black circle symbolizes the outliers.

**Figure 13:**
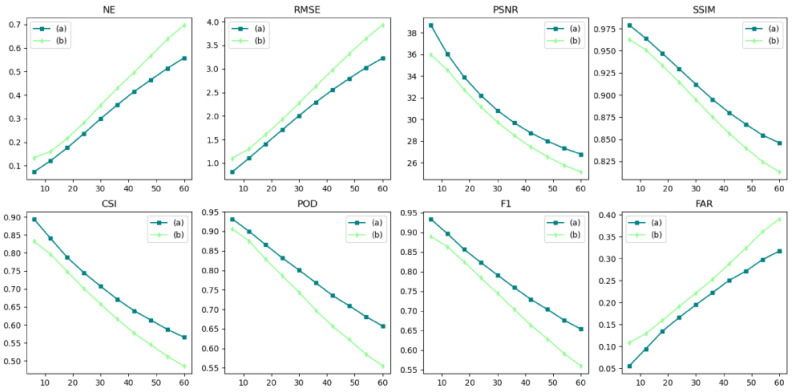
The forecast effect at future moments, (a) and (b) representing AANet and NowcastNet, respectively.

The forecasted performance of AANet and NowcastNet is verified and the forecasted results are shown in Figure 11, Figure 12 and Figure 13, Table 3.

As shown in Figure 11, the forecasted effect of AANet is better than the forecasted effect of NowcastNet. As the number of time steps increases, NowcastNet’s forecast gradually converge to the mean (using the mean to cover all details), but AANet provides more accurate forecast (showing the more details).

Box plot of model forecasted effect as shown in Figure 12. AANet vs. NowcastNet: for NE, RMSE, and FAR, the median line of AANet and the variance of AANet are smaller than the median line of NowcatNet and variance of NowcatNet; for PSNR, SSIM, CSI, POD, and F1, the median line of AANet is higher than the median line of NowcastNet, and the variance of AANet is smaller than the variance of NowcatNet. As shown in Figure 13, the forecasted performance of AANet is better than NowcastNet in all future moments. AANet compared to NowcastNet: for NE, RMSE, and FAR, AANet is smaller than NowcastNet in all future moments; for PSNR, SSIM, CSI, POD, and F1, AANet is greater than NowcastNet in all future moments. Since AANet uses the autoregressive method (A), SSIM Loss, and the two-stage adversarial loss. The autoregressive method (A) only suffers from the “regression to average” issue; SSIM Loss suppresses the “regression to average” issue; the two-stage adversarial loss allows the generator to produce more realistic and more similar provisional and final forecasted data. Because NowcastNet uses the evolutionary method (E) and an imperfect adversarial strategy (Generating data and observational data are defined as true data, ignoring the significance of provisional generated data.), NowcastNet accumulates excessive errors and mistakenly defines generating data as true data.

As shown in Table 3, AANet vs. NowcastNet, the performance of AANet is superior: 0.0763 reduced in NE, 0.377 reduced in RMSE, 1.45 improved in PSNR, 0.0208 improved in SSIM, 5.78% improved in CSI, 6.25% improved in POD, 5.7% improved in F1, and 4.2% reduced in FAR. Because the autoregressive method (A) and SSIM Loss reduce the excessive errors, the NE, RMSE and FAR of AANet are smaller than the NE, RMSE and FAR of NowcastNet. Becuase the two-stage adversarial loss promotes AANet’s generator to generate more realistic and more similar provisional and final forecasted data, and the provisional forecasted data promotes AANet’s second-stage generator to generate more similar and more realistic final forecasted data. AANet’s PSNR, SSIM, CSI, POD, and F1 are greater than NowcastNet’s PSNR, SSIM, CSI, POD and F1. In summary, the forecasted performance of AANet is better than NowcastNet.

#### 3.3.4. AANet with or without Adversarial Strategies

**Figure 14:**
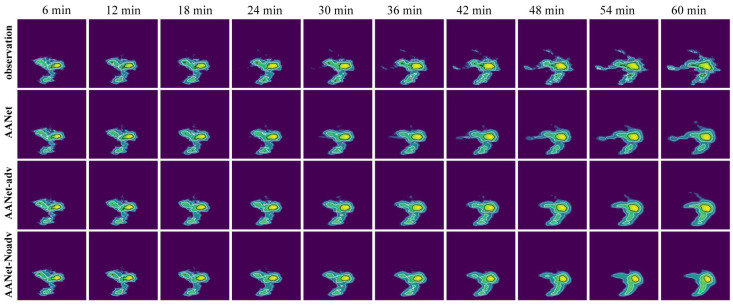
Visualization of the forecast effect; the contour lines (light blue < white < black) are gradient lines of precipitation intensity.

**Figure 15:**
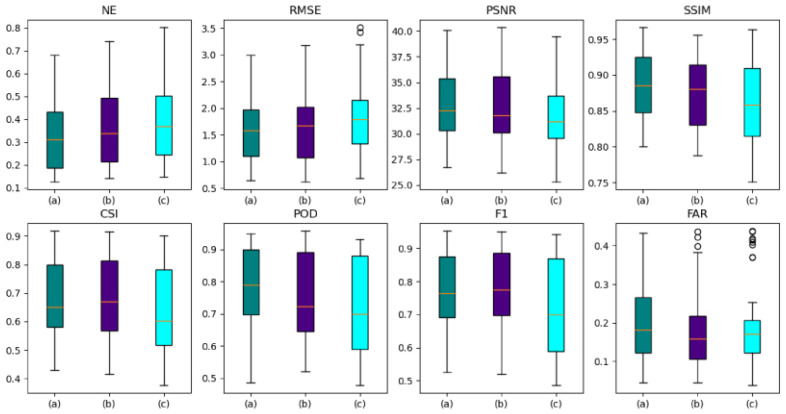
Box plot of model forecasted effect, among which (a), (b), and (c) represent AANet, AANet-adv, and AANet-Noadv, respectively. The orange line represents the median line, and the black circle symbolizes the outliers.

**Figure 16:**
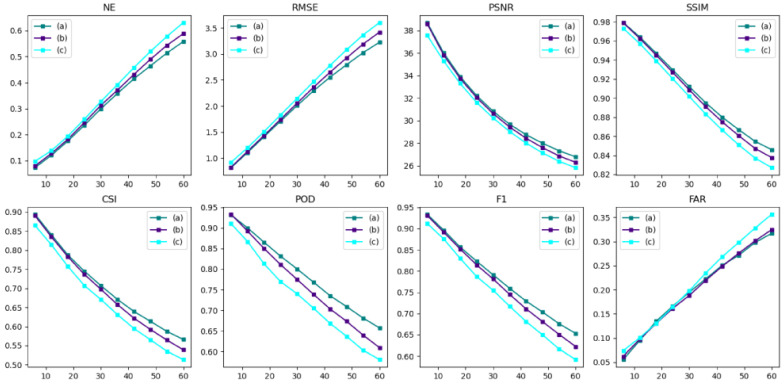
The forecast effect at future moments, (a), (b), and (c) represent AANet, AANet-adv, and AANet-Noadv, respectively.

Verify whether the two-stage adversarial strategy works positively by comparing AANet, AANet_adv (the traditional generative adversarial strategy with no use of provisional forecasted data in the adversarial stage), and AANet_Noadv (no use of adversarial strategy), with the forecasted effects as shown in Figure 14, Figure 15 and Figure 16, Table 4.

As shown in Figure 14, the adversarial strategy acts positively on AANet. As the number of future moments increases, the adversarial strategy gradually takes effect, enhancing the model’s ability to fake. Since AANet of the two-stage adversarial strategy is better than AANet of the traditional adversarial strategy, AANet of the two-stage adversarial strategy provides more realistic pseudo-data.

A box plot of model forecasted effect as shown in Figure 15. Although the adversarial strategy exacerbates the model’s FAR, the adversarial strategy improves model’s other performance. For both NE and RMSE, the adversarial strategy effectively reduces the errors in the forecasted data, whereas the two-stage adversarial strategy outperforms the traditional adversarial strategy; for PSNR, SSIM, and POD, the adversarial strategy effectively improves the accuracy of the forecasted data, in which the two-stage adversarial strategy is significantly better than the traditional adversarial strategy; for CSI and F1, the adversarial strategy effectively improves the accuracy of the forecasted data, where the traditional adversarial strategy outperforms the two-stage adversarial strategy.

As shown in Figure 16, the adversarial strategy plays a positive role in the forecasted performance of AANet. As the future moments increase, the broken lines of AANet and AANet-adv are gradually separated, and the two-stage adversarial strategy acts negatively on the decay rate of the forecasted performance (better than the traditional adversarial strategy), which effectively suppresses the weakening of the forecasted performance.

As shown in Table 4, the adversarial strategy plays a positive role in the forecasted performance, with AANet having the best forecast, AANet-adv having the second best, and AANet-Noadv having the worst. The AANet with traditional adversarial strategy (AANet-adv) outperforms the AANet without adversarial strategy (AANet-Noadv), where NE is reduced by 0.0223, RMSE is reduced by 0.112, FAR is reduced by 1.36, PSNR is improved by 0.49, SSIM is improved by 0.0076, CSI is improved by 2.48, POD is improved by 3.19, and F1 is improved by 2.54. AANet outperforms AANet with traditional adversarial strategies (AANet-adv), where NE is reduced by 0.0157, RMSE is reduced by 0.083, FAR is reduced by 0.13, PSNR is improved by 0.29, SSIM is improved by 0.0042, CSI is improved by 1.47, POD is improved by 2.66, and F1 is improved by 1.52. The traditional adversarial strategy promotes the generator to produce more realistic and more similar final forecasted data, and the two-stage adversarial strategy promotes the generator to produce more realistic and more similar final forecasted data through more realistic and more similar provisional forecasted data.

In summary, both the two-stage adversarial strategy and the traditional adversarial strategy play a positive role in the model’s forecasted performance, but the two-stage adversarial strategy is the most effective.

#### 3.3.5. Ablation Experiments of AANet

As shown in Table 5, ablation experiments are performed on AANet. FURENet is the first-stage generative model of AANet, Semantic Synthesis Model (SSM) is the second-stage generative model of AANet. SSIM Loss, Tadv Loss, and adv Loss are all loss functions (Tadv Loss is the two-stage adversarial loss, and adv Loss is the traditional adversarial loss).

FURENet suffers from the “regression to average” issue; thus, FURENet can only generate ambiguous forecasted data. SSIM Loss suppresses the “regression to average” issue; thus, SSIM Loss has a positive effect on FURENet. The main task of SSM is to reprocess the generated data of FURENet (NowcastNet uses a second-stage model to process the generated data of UNet), but SSM acts negatively on FURENet due to multi-stage models’ easily accumulated errors. SSIM Loss reduces the errors that the multi-stage model accumulates, but the forecasted effect is not as good as the combination of the FURENet and SSIM Loss. Both the traditional adversarial strategy and the two-stage adversarial strategy work positively on the multi-stage model, but the two-stage adversarial strategy is superior. The two-stage adversarial strategy is superior to the traditional confrontation strategy: traditional adversarial strategies only uses the final generated data but ignores the importance of the provisional generated data (the first-stage model is gradient separated from the second-stage model); with the two-stage adversarial strategy, the first-stage model generates more similar and more realistic provisional forecasted data, and the second-stage model generates more similar and more realistic final forecasted data through using the temporary forecast data. If the parameters α and β of Equation (27) are set to 1 and 4, AANet achieves even better results with NE = 0.2772, RMSE = 1.893, PSNR = 31.91, SSIM = 0.9177, CSI = 72.70%, POD = 79.97%, F1 = 80.16%, and FAR = 17.36%.

## 4. Conclusions

The previous nowcasted deep learning models have the “regression to average” issue and cannot provide a complete generative adversarial strategy. In this study, the Adversarial Autoregressive Network (AANet) is proposed, which uses the structural similarity loss (SSIM Loss) function and proposes Two-stage Adversarial Loss (Tadv Loss), and the dataset is “NJU_CPOL_update2308”. To verify the effect of SSIM Loss and Tadv Loss, AANet is compared with the current nowcasted model in a comparative experiment. The results show that SSIM Loss reduces the impact of the “regression to average” issue, and Tadv Loss reduces the errors that the multi-stage model accumulates and promotes the model to generate more realistic false data in both stages. SSIM Loss and Tadv Loss help our model outperform the baseline model regarding forecasted effectiveness. The main contributions of this study are as follows:

1. The previous model employed multiple convolutional neural networks to generate future fields and implicitly and iteratively evolved them to produce forecasted data. Nevertheless, convolutional neural networks are afflicted by the “regression to average” issue, and simple iterative evolution is susceptible to errors. Therefore, the generation is directly the forecasted data via FURENet, and the SSIM Loss is employed to mitigate the impact caused by the “regression to the mean” issue.
2. The previous model adopted multi-stage generation to forecasted data (first generating provisional forecasted data and then generating the final forecasted data) and calibrated the final forecasted data with the help of a discriminator. However, it ignored the importance of the provisional forecasted data and failed to reduce the errors that the multi-stage model accumulates. Therefore, Tadv Loss is used to reduce the errors that the multi-stage model accumulates and enhances the forecasted performance in two stages.

Therefore, this study provides a feasible and forecasted solution based on generative adversarial networks and dual-polarization radar observations.

Limitations of this work: The model does not provide forecasted data of the next 2–3 h, and the dataset lacks radar observations of longer duration.

Future work outlook: Based on deep learning methods and spatial affine trans-formations, we research a forecasted model that can provide longer-term (2–3 h) forecasted data. We work together with the local weather bureau to obtain a larger sample of data from the same geographic area and employ AANet in the domain of temperature prediction for forecasting the temperature in the future period.

## Figures and Tables

**Table 1:** The table presenting the average values of indicators for the forecasted methods (ranging from 6 min to 60 min).

	NE	RMSE	PSNR	SSIM	CSI	POD	F1	FAR
E-FURENet	0.4606	2.780	28.78	0.8740	61.31	70.79	69.45	27.30
A-FURENet	0.3452	2.223	30.83	0.9005	68.47	75.39	76.13	20.34
E-UNet	0.4906	2.870	28.56	0.8683	60.03	68.69	68.18	26.23
A-UNet	0.3486	2.307	30.62	0.8976	66.86	72.15	74.43	19.71

**Table 2:** The table presenting the average values of indicators for the forecasted methods (ranging from 6 min to 60 min).

	NE	RMSE	PSNR	SSIM	CSI	POD	F1	FAR
A-FURENet	0.3452	2.223	30.83	0.9005	68.47	75.39	76.13	20.34
A-FURENet_NS	0.3559	2.228	30.68	0.8961	67.94	75.24	75.56	21.31
A-UNet	0.3486	2.307	30.62	0.8976	66.86	72.15	74.43	19.71
A-UNet_NS	0.3648	2.315	30.42	0.8932	66.39	72.82	73.90	21.81

**Table 3:** The table presenting the average values of indicators for the forecasted methods (ranging from 6 min to 60 min).

	NE	RMSE	PSNR	SSIM	CSI	POD	F1	FAR
AANet	0.3216	2.088	31.24	0.9075	70.63	78.92	78.31	19.96
NowcastNet	0.3979	2.465	29.79	0.8867	64.85	72.67	72.61	24.16

**Table 4:** The table presenting the average values of indicators for the forecasted methods (ranging from 6 min to 60 min).

	NE	RMSE	PSNR	SSIM	CSI	POD	F1	FAR
AANet	0.3216	2.088	31.24	0.9075	70.63	78.92	78.31	19.96
AANet-adv	0.3373	2.171	30.95	0.9033	69.16	76.26	76.79	20.09
AANet-Noadv	0.3596	2.283	30.46	0.8957	66.68	73.07	74.25	21.45

**Table 5:** The table presenting the average values of indicators for the forecasted methods (ranging from 6 min to 60 min).

FURENet	SSM	SSIM Loss	Tadv Loss	adv Loss	NE	RMSE	PSNR	SSIM	CSI	POD	F1	FAR
√					0.3559	2.228	30.68	0.8961	67.94	75.24	75.56	21.31
√		√			0.3452	2.223	30.83	0.9005	68.47	75.39	76.13	20.34
√	√				0.3724	2.308	30.14	0.8936	66.12	72.91	73.76	22.53
√	√	√			0.3596	2.283	30.46	0.8957	66.68	73.07	74.25	21.45
√	√	√		√	0.3373	2.171	30.95	0.9033	69.16	76.26	76.79	20.09
√	√	√	√		0.3216	2.088	31.24	0.9075	70.63	78.92	78.31	19.96

## Data Availability

Dataset available on request from the authors.
